# Non-English primary language and disparities in stroke outcomes after mechanical thrombectomy: a single institution study

**DOI:** 10.3389/fstro.2023.1224566

**Published:** 2023-07-05

**Authors:** Nurose Karim, Suzanne Stone, Amber Salter, Mehari Gebreyohanns, Mark Johnson, Erica Jones

**Affiliations:** ^1^Department of Neurology, University of Texas Southwestern Medical Center, Dallas, TX, United States; ^2^Department of Neurology, Statistical Planning and Analysis Section, University of Texas Southwestern Medical Center, Dallas, TX, United States

**Keywords:** disparities, language, acute care, thrombectomy, emergency medicine

## Abstract

**Background:**

Delays in acute treatment of ischemic stroke have been associated with worse outcomes. While having a non-English primary language has not been shown to delay receiving thrombolytic therapy, we assessed whether non-English primary language was associated with worse functional outcomes in patients receiving mechanical thrombectomy (MT).

**Method:**

This is a retrospective study of our MT database from two comprehensive stroke centers from January 2016 to May 2021. Primary endpoint was discharge modified Rankin Scale (mRS) 0-2. Differences between English primary language (EPL) and non-English primary language (nEPL) groups were evaluated using an analysis of variance (ANOVA), Kruskal-Wallis and chi square test. Multivariable logistic regression was used to evaluate EPL vs. nEPL patients using data driven models determined by stepwise selection approach.

**Result:**

We identified 276 patients receiving MT with 83% EPL and 17% nEPL patients. nEPL patients had higher mean hemoglobin A1c, were less likely to have insurance, and more likely to have symptomatic intracranial hemorrhage compared to EPL patients (Table). We observed a longer median ED arrival to groin puncture time in the nEPL group. No differences were observed in discharge or mRS 0-2 in the univariate or multivariable logistic regression.

**Discussion:**

Despite finding longer ED length of stay among nEPL patients, there was no difference between nEPL and EPL in good functional outcome rates in patients treated with MT.

## Introduction

Acute ischemic stroke is a recognized neurological emergency. Delays in care can have a significant impact on functional or mortality outcomes and efforts to improve outcomes often focus on reducing delays in care. There has been an increasing focus on racial/ethnic disparities in stroke outcomes and more studies have sought to identify the sources of these differences. Certain patient characteristics that may differ by race/ethnicity such as access to emergency medical services (EMS) or insurance status have been shown to contribute to delays in care (Smith et al., 2010). Other patient characteristics such as cultural background and primary spoken language may themselves also contribute to disparity in access to care. Patients with limited English proficiency represent an 'at risk' group in our healthcare system (Litzau et al., 2018). According to 2018 Census Bureau, 22% of the U.S. population speak a language other than English as their primary language. The second most common language spoken in the U.S. is Spanish (13.5%) followed by Asian and Pacific languages (3.6%) (Bureau, 2004). In Texas, approximately 65% of the population speak only English, 35% speak a language other than English, and 29% speak Spanish as a primary language (Bureau, 2013). Even with interpretation services available, communication can be limited or delayed during an acute emergent presentation (Solomon et al., 2020) especially among stroke patients presenting with alteration of consciousness or impaired language functions.

There have been previous studies on the effect of limited English proficiency (LEP) on treatment times in IV thrombolysis in patients with acute ischemic stroke, however, the results have been inconsistent. These studies have found a variety of effects of LEP on care, ranging from no significant effect to a longer door to needle (DTN) time, or significant increase in the median onset to needle time in patients with LEP (Dujari et al., 2016; Rezania et al., 2018; Anderson et al., 2020). Cauchi et al. (2017) in their study of 209 patients who received intravenous thrombolytics, found that among Hispanic patients, 17% had an average of 12-minute delay in door to needle time attributed to language barrier. Recent studies on racial disparities among stroke patients receiving mechanical thrombectomy have shown worse door to puncture times for Hispanic patients where language barriers potentially contributed to the delays but were not directly studied (Jones et al., 2021; Siegler et al., 2022). Many stroke patient registries do not collect spoken language or level of English proficiency data. As far as these authors could ascertain, there have been no studies directly examining the impact of language barrier, rather than ethnicity, on delays in mechanical thrombectomy (MT) treatment metrics or outcomes. We hypothesized that patients with LEP that are eligible for mechanical thrombectomy could have worse functional outcomes at discharge due to delays in early assessment.

## Methods

This is a retrospective observational study of our MT database from our institution's two comprehensive stroke centers in Dallas, TX from January 2016 to May 2021. Both centers within our institution are overseen by the same Neurology and Endovascular faculty and trainees. The centers operate under similar stroke treatment protocols and are located within a mile of each other in the Medical District in Dallas County. Using data from both hospital sites increased the inclusion of patients from different economic backgrounds which could reduce selection bias. Patients presented to our ED via ambulance or private transport, were identified as a code stroke and evaluated per our institution's standard stroke protocol including assessment for mechanical thrombectomy eligibility per our imaging protocol. Patient outcomes and demographic data, such as primary language, were collected prospectively as a part of our stroke patient registries at each hospital from the EMR. Missing data was abstracted from the electronic medical record by members of the stroke team where possible and was otherwise noted as missing (Table 1).

**Table 1:** Demographics and risk factors.

	**English primary language (*N =* 229)**	**Non-English primary language (*N =* 47)**
**Demographics**		
Age	66.7 (14.3)	64.6 (16.1)
Female	116 (50.7)	23 (48.9)
Male	113 (49.3)	24 (51.1)
White	135 (65.9)	31 (68.9)
African American	67 (32.7)	3 (6.7)
Asian	2 (0.98)	6 (13.3)
Hispanic ethnicity	26 (11.5)	37 (80.4)
Insurance	178 (84.0)	18 (40.0)
Medicare	106 (47.1)	28 (60.9)
**Medical history**		
Prior stroke	35 (15.8)	8 (17.8)
HTN	193 (84.3)	36 (76.6)
Diabetes mellitus	85 (37.3)	26 (55.3)
HA1c	6.2 (1.7)	7.0 (2.3)
Tobacco abuse	68 (30.1)	8 (17.8)
**Pre-morbid mRS**		
• 0	116 (58.9)	27 (61.4)
• 1	39 (19.8)	10 (22.7)
• 2	20 (10.2)	3 (6.8)
• 3	14 (7.1)	3 (6.8)
• 4	6 (3.0)	1 (2.3)
• 5	2 (1.0)	0 (0.0)
**Treatment**		
Location: Hospital 1	54 (23.6)	33 (70.2)
Location: Hospital 2	175 (76.4)	14 (29.8)
LKN to arrival time (min)	244.0 [84.0,513.0]	198.0 [76.5,605.0]
LKN to groin puncture (min)	353.5 [203.5,642.0]	338.0 [191.0,787.0]
ED arrival to groin puncture (min)	83.0 [42.5,146.5]	112.0 [84.0,154.0]
Groin Puncture to Revascularization (min)	56.0 [33.0,83.0]	46.5 [36.0,71.5]
Arrival via EMS	101 (45.1)	24 (53.3)
Transfer from outside hospital	98 (43.8)	6 (13.3)
Private transport from home	22 (9.8)	13 (28.9)
Inpatient stroke	3 (1.3)	2 (4.4)
tPA given	86 (37.7)	22 (46.8)
NIHSS on admission	16.0[10.0,20.5]	16.0[9.0,22.0]
Hemorrhagic transformation	30 (13.1)	12 (26.1)
TICI 2B/3 reperfusion	198 (88.0)	37 (80.4)

Values presented as Mean ± SD, Median [P25, P75] or N (column %).

For our analysis, only patients who underwent mechanical thrombectomy for acute stroke were collected from the registry. Patients were categorized as non-English primary language (nEPL) in the registry if a language other than English was listed as the preferred language in their electronic record for the admission or English primary language (EPL). The specific language was not recorded during data abstraction, so we were unable to comment on different primary language subsets. Patients with incomplete data on primary outcome measure were excluded. Primary endpoint was a discharge modified Rankin Scale (mRS) 0-2 (binary outcome). Secondary outcomes were 90-day mRS 0-2 (binary), 90-day mRS (ordinal), discharge mRS (ordinal), discharge National Institute of Health Stroke Scale (NIHSS). Utilization of interpreter service is established practice in the ED is standard practice in both centers when fluent and certified medical personnel are unavailable. Utilization is not recorded consistently in the electronic medical record during the period of the study.

### Statistics

Descriptive statistics were used to summarize the demographic and clinical characteristics such as age, race, ethnicity, medical history, time metrics, NIHSS and treatments administered. Means/standard deviation or median/25th and 75th percentiles for continuous variables and frequency/percentages for categorical variables. Differences in demographic and clinical characteristics between English primary language (EPL) and non-English primary language (nEPL) patients were evaluated using an analysis of variance (ANOVA), Kruskal-Wallis and chi square test, as appropriate. Multivariable logistic regression was used to evaluate differences in the primary endpoints between EPL vs. nEPL patients. The multivariable model used a stepwise selection approach with variable selection based on the corrected Akaike information criterion (AICC). Odds ratios (OR) and 95% confidence intervals (95%CI) are reported. The significance level was set at 0.05. The senior author had full access to the data and is responsible for data integrity and data analysis.

## Results

We identified 276 patients receiving mechanical thrombectomy within our stroke registry who were included in the analysis. The average age of our sample was 66 with approximately equal men and women represented. The sample was 66% White, 28% African American, 3% Asian, and 2% Hispanic. Patient mode of presentation varied with 47% arriving via ambulance, 39% transferring from outside hospital, and 13% arriving via private vehicle. The average last known normal to ED arrival time was 234 min, or almost 4 h. 39% were treated with thrombolytics prior to mechanical thrombectomy. The average NIHSS on arrival was 16. The rate of TICI 2b/3 reperfusion was 87 and 15% of patients developed hemorrhagic transformation. We found 83% of these patients were EPL and 17% were nEPL patients. Among nEPL patients, 69% identified as White, 11% as Hispanic, 7% as African American, and 13% as Asian.

**Figure 1:**
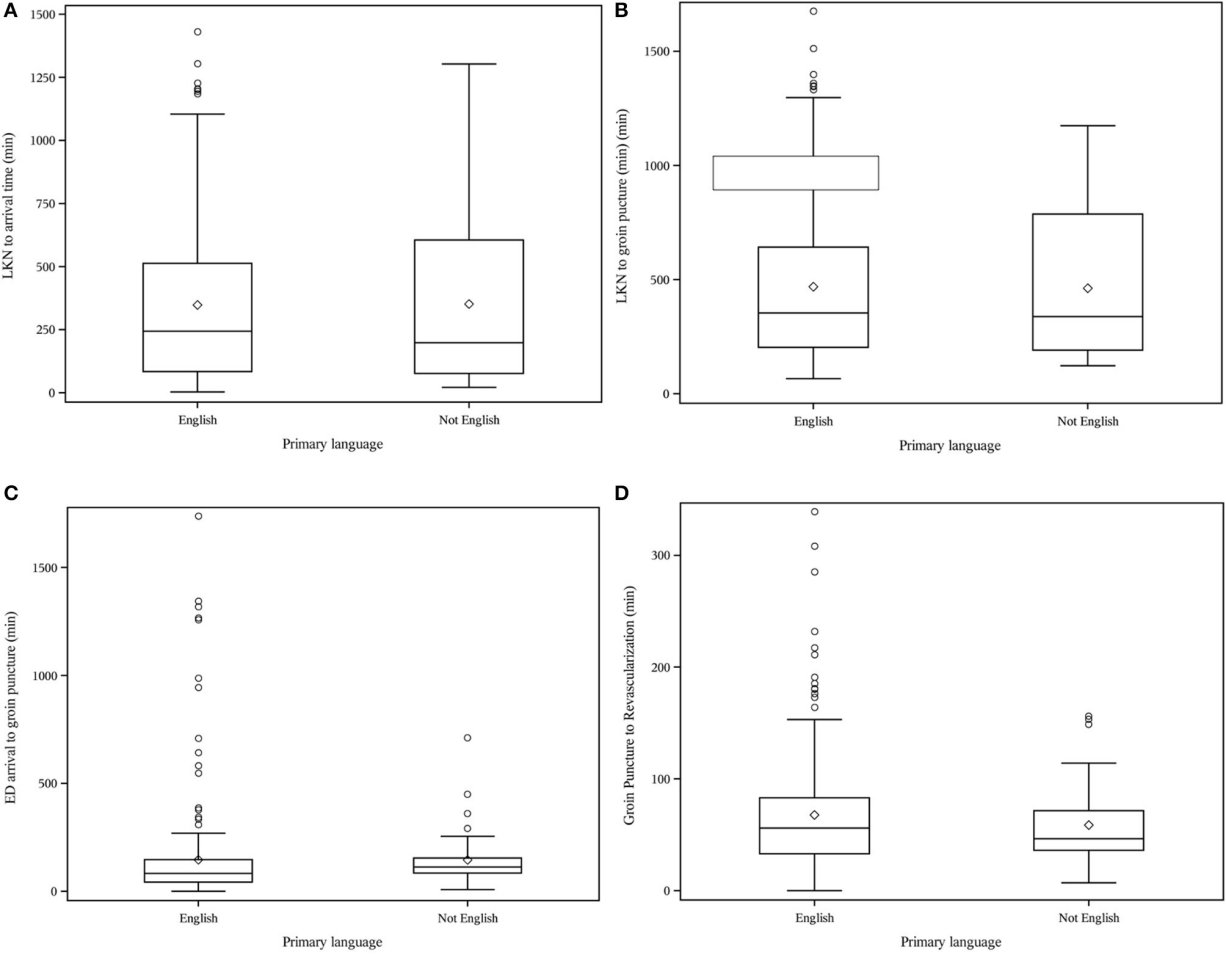
**(A)** LKN to arrival time (min), **(B)** LKN to groin puncture (min), **(C)** ED arrival to groin puncture (min), **(D)** Groin Puncture to Revascularization (min).

Compared to the EPL group, nEPL patients were less likely to be insured (40 vs. 84%, *p* < 0.001), presented with higher mean hemoglobin A1c (7.0 vs. 6.2, *p* = 0.008) and higher rates of diabetes mellitus (55 vs. 37%, *p* = 0.02, Table 1). Age, gender, rates of hypertension, tobacco abuse and premorbid mRS were not statistically significantly different between the nEPL and EPL groups. Mode of arrival varied between the groups (*p* < 0.001); 53% nEPL patients arrived by EMS vs. 45% in the EPL group, 13% nEPL were transferred from outside hospital vs. 43% EPL patients, 29% nEPL group arrived via private vehicle vs. 10% EPL group. Stroke severity defined as median NIHSS on admission was similar between groups. nEPL patients were also more likely to have intracranial hemorrhagic transformation (26 vs. 13%, *p* = 0.025) compared to EPL patients but had similar rates of successful reperfusion (Table 1). In treatment time metrics (Figure 1), we observed a significantly longer median ED arrival to groin puncture time in the nEPL group (112 vs. 83 min, *p* = 0.02). No differences were found in other time metrics such as last known normal (LKN) to ED arrival time or LKN to groin puncture time.

The primary outcome showed no statistically significant difference in rates of discharge mRS 0-2 (EPL 24.6 vs. nEPL 13.6%) (Table 2) with unadjusted OR 0.49 95% CI (0.18–1.13) and adjusted OR 0.55 95% CI (0.19–1.39). No differences were observed in rates of 90-day mRS 0-2 (EPL 34 vs. nEPL 30%) in unadjusted OR 0.83 95% CI (0.40–1.66) or adjusted 1.23 95% CI (0.49–3.02) analysis. Discharge mRS data was missing for 8 patients and 90-day mRS was missing for 23 patients. Median discharge NIHSS was the same between both groups at 8.0 [unadjusted OR 1.00 95% CI (0.98–1.03)].

**Table 2:** Discharge outcomes by language group.

	**English primary language (*N =* 229)**	**Non-English primary language (*N =* 47)**	***p*-value**	**Odds ratios OR (95% CI)**	**Adjusted odds ratio OR (95% CI)**
Discharge mRS category			0.11		
>2	169 (75.4)	38 (86.4)		Reference	
0–2	55 (24.6)	6 (13.6)		0.49 (0.18, 1.13)	^a^0.55 (0.19, 1.39)
Discharge modified Rankin	4.0 (3.0, 5.0)	4.0 (3.0, 5.0)	0.19	-	
Discharge NIHSS	8.0 (2.0, 16.0)	8.0 (3.0, 20.0)	0.61	-	
90-day mRS category			0.61		
>2	138 (65.7)	30 (69.8)		Reference	
0–2	72 (34.3)	13 (30.2)		0.83 (0.40, 1.66)	^b^1.23 (0.49, 3.02)
90 day modified Rankin	4.0 (2.0, 5.0)	4.0 (2.0, 6.0)	0.91		

^a^Discharge mRS adjusted for admission NIHSS and hemorrhagic transformation.

^b^90 day mRS Adjusted for age, hemoglobin A1c, admission NIHSS, hemorrhagic transformation, and TICI 2B/3 reperfusion.

## Discussion

In this study, our objective was to determine if primary spoken language had an impact on outcomes in our patients requiring mechanical thrombectomy. We found certain baseline demographic differences in medical history and mode of presentation between our EPL and nEPL speaking groups which were similar to what has been reported in a previous study (Smith et al., 2010). Based on mixed results from previous studies on treatment with thrombolysis, we anticipated that nEPL could have a role in delaying evaluation for mechanical thrombectomy leading to worse outcomes. Interestingly, we found no difference in time from LKN to ED arrival despite differences in mode of transportation between the groups. While there was no difference in overall LKN to revascularization time, there was a significant difference in median ED arrival to groin puncture time which was 29 min longer in the nEPL group (Figure 1). Without more detailed information on the ED workflow, we cannot easily identify the cause for this delay. Consenting is completed in-person or by phone and could have been delayed by interpreter utilization. There is also potential for delays in recognition of stroke among nEPL patients due to poor communication of symptoms or onset time with the ED staff. Ultimately, despite longer ED length of stay, there was no difference between nEPL and EPL groups in the primary outcome of discharge mRS 0-2.

In the existing stroke literature, it is well established that earlier treatment with IV thrombolysis improves the chances of functional recovery in stroke patients (Lees et al., 2010). There have been a few previous studies on language disparity and acute stroke care with thrombolysis. Rostanski et al. (2016) investigated the language discordance between patients and physicians and found no effect on door-to-imaging time and door-to-needle time. A single center analysis of 3894 acute ischemic stroke patients found no significant difference in intravenous thrombolysis treatment rate between English and non-English-preferring patients (Luan Erfe et al., 2016). While most studies have not shown much effect of language barrier on acute stroke treatment metrics, stroke outcomes studies have shown conflicting results. A study of the Registry of the Canadian Stroke Network showed reduced mortality and better performance on some quality-of-care measures in patients with language barriers (Shah et al., 2015). Data from the Australian Stroke Clinical Registry, however, showed that patients requiring interpreters had comparable discharge outcomes but poorer health-related quality of life compared to patients not requiring interpreters (Kilkenny et al., 2018). The effect of language barrier on ED length of stay has also been evaluated in non-stroke populations where critically ill, non-English speaking patients were reported to spend an average of 17–35 more minutes in the ED before arriving to ICU leading to higher mortality rates (Oca et al., 2021).

All healthcare institutes that receive federal funds are already required to provide qualified medical interpreters to patients with LEP (USDHHS, 2017). Karliner et al. (2007) performed a systematic literature search to understand the impact of professional and ad hoc interpreters on clinical care in patients with LEP. The study concluded that the presence of professional interpreters is associated with a decrease in communication errors, increases patient comprehension, equalizes health care utilization, improves clinical outcomes, and increases patient satisfaction with communication and clinical services. Joseph et al., in their Australian health system analysis, found no difference in patients' satisfaction between in-person, video, telephone interpretation or interpretation provided by the treating bilingual physician (Joseph et al., 2018). Also, it has been suggested to increase the number of qualified bilingual in-house staff, as for example, by enrolling residents in Clinician Cultural and Linguistic Assessment (CCLA) certification program (Solomon et al., 2020). Through this initiative, their newly certified residents could translate languages spoken by nearly 250 of their patients in the hospital annually. Clear and standardized translations for key stroke care terminology in the patient's primary language, as was demonstrated in the impact of creating a standard term for “stroke” in Amharic, can also facilitate understanding during interpretation (Aseffa et al., 2019). Potential contributors to the delay seen in our population, could be due to non-familiarity or discomfort of the ED physicians to recognize stroke in patients with LEP.

A portion of our study was conducted during peaks in the COVID pandemic. We did not have patient COVID status collected in our data, which is a limitation, and it was unclear how this variable may impact our analysis given overall delays in emergency department care during portions of the pandemic. Additional limitations of our study include our small sample size and relatively small numbers of nEPL speakers which make it difficult to identify significant differences between the nEPL and EPL groups. Primary language designation does not indicate that nEPL patients could not have high English proficiency. Our registry did not collect the level of English proficiency so that we could account for bilingualism in the analysis. The majority of our nEPL patients are Spanish speaking in our centers, but we noted that Ethnicity category was not related to nEPL and only a small percentage identified with Hispanic ethnicity. We collected self-reported ethnicity data and many of the nEPL subjects self-identified as White race with or without Hispanic ethnicity demonstrating that race/ethnicity cannot be conflated with language.

## Conclusions

For many counties in the U.S., the percentage of the population with non-English primary language is growing and is a possible source of widening disparities if steps are not taken to ensure that these patients receive equal access to care. For future study, a larger sample size and multicenter participation would increase the likelihood of identifying the smaller effects of language barriers on clinical outcomes after mechanical thrombectomy. More detailed data on evaluation processes in the ED would clarify where delays may occur and allow for adjustment of protocols that improve patient flow through the ED. Future studies may also show the impact of availability of interpreter services and multilingual staff on acute stroke care in this population. These studies would inform interventions to improve stroke health equity by improving access to care for a growing demographic group.

## Data availability statement

The raw data supporting the conclusions of this article will be made available by the authors, without undue reservation.

## Author contributions

NK and EJ contributed to conception and design of the study. SS organized the database. AS performed the statistical analysis. NK wrote the first draft of the manuscript. NK, AS, and EJ wrote sections of the manuscript which were revised by MJ and MG. All authors contributed to manuscript revision, read, and approved the submitted version.
